# Case of Hypercalcemia Secondary to Hypervitaminosis A in a 6-Year-Old Boy with Autism

**DOI:** 10.1155/2011/424712

**Published:** 2011-09-11

**Authors:** Arpita Kalla Vyas, Neil H. White

**Affiliations:** Division of Pediatric Endocrinology and Diabetes, Department of Pediatrics, Washington University in Saint Louis and Saint Louis Children's Hospital, Saint Louis, MO 63110, USA

## Abstract

Vitamin A intoxication secondary to over-the-counter nutritional supplements and from its use in acne treatment has been described. However, there have been very few case reports of chronic hypervitaminosis A leading to hypercalcemia in the pediatric population. This paper describes a boy with hypercalcemia secondary to chronic vitamin A intoxication in the context of vitamin A usage for therapy of autism. In addition to discontinuation of vitamin A, hyperhydration, and furosemide, the hypercalcemia in this patient required the use of prednisone and pamidronate to normalize the calcium.

## 1. Introduction

Hypercalcemia is an infrequent occurrence in children except during the initial newborn period. The various etiologies of hypercalcemia are also relatively rare in children. In this paper, we describe a 6-year-old autistic boy who developed hypercalcemia secondary to hypervitaminosis A, and we review the literature related to hypervitaminosis A and hypercalcemia.

## 2. Case Report

A 6-year-old boy presented to our emergency room because of vomiting and abdominal pain. His parents reported that he had a 3-day history of low-grade fever together with abdominal pain, emesis, and constipation. There was no history of altered mental status, coryzal symptoms, or headaches. He was reported to have “autism”, and four weeks earlier he had been placed on a “specific carbohydrate diet” including “egg protein shakes.” Two days later, he developed “hives”, and the egg protein shakes were discontinued. However, pruritus continued and he developed excoriated and dry lips over the next few days. These latter symptoms continued until his presentation to our emergency room. 

His past medical history was significant for premature birth at 30 weeks gestation, nocturnal enuresis for the past 8 months with some recent improvement using a bed alarm, and an inguinal hernia repair at the age of 3 years. He had been diagnosed with pervasive behavioral/developmental disorder attributed to autism. In an attempt to manage his behavior, he was started on a casein- and gluten-free “specific carbohydrate diet” (eggs, fruits, vegetables without complex carbohydrates) together with protein shakes. These had been discontinued two weeks prior to admission because of the development of a skin rash and pruritis. He had also been placed on multiple dietary and vitamin supplements as part of the management of his autism. These included 6 drops/day of vitamin A (equivalent to 30,000 IU per day) added to his diet for the past 4 months, Vitamin B12 shots twice per week, a custom-made multivitamin, Vitamin E Drops, Coenzyme Q and Omega-3 fatty acids. 

Family history revealed that his father had a history of kidney stones as a young adult, which had not been further evaluated and have not recurred. Maternal grandmother had thyroid surgery for an “overactive thyroid.” There was no family history of hyperparathyroidism, thyroid neoplasms, pheochromocytomas, pituitary tumors, or islet cell tumors. 

On examination when he was initially seen in the emergency unit, his weight was 21.6 kg (50th percentile) and height 123.5 cm (80th percentile). He was sleepy and in discomfort complaining of abdominal pain. He was mildly dehydrated. He was noted to have cheilitis and a blanching erythematous rash on his face. He had symmetric hyperreflexia in all four limbs with normal muscle tone. Funduscopic examination was within normal limits. HEENT, abdominal, cardiovascular, and respiratory exam were all within normal limits. There was no evidence of thyromegaly, and thyroid gland was nontender. Sexual maturation was Tanner Stage 1. 

On initial presentation, he had a normal basic metabolic profile except that the total calcium was 13.7 mg/dL (normal range: 8.6–10.3). Serum creatinine was 0.6 mg/dL and BUN 26 mg/dL indicative of his mild dehydration. Intravenous hydration was initiated, and he was hospitalized for evaluation and treatment of hypercalcemia. He was initially treated with intravenous hydration using 0.9% saline at 3 L/m^2^/day. He also received a single dose of intravenous furosemide 1 mg/kg. His calcium stabilized with hydration and furosemide therapy within 48 hours and remained stable (though still near or slightly above the upper limits of normal) off IV fluids for 24 hours (see Table 1). 

Supplemental vitamin A was discontinued on admission. During his initial hospitalization, serum phosphorus was 4.0 mg/dL (normal: 3.0–6.0 mg/dL), intact PTH <3 pg/mL (normal: 14–72), 25-hydroxy-vitamin D 22.3 ng/mL (normal: 10–55), and 1,25 dihydroxy-vitamin D 16 pg/mL (normal: 22–67). TSH 2.29 mcIU/mL (normal: 0.35–5.5), Free T4 0.95 ng/dL (normal: 0.9–1.8 ng/dL), alkaline phosphatase 209 IU/L (normal: 140–420), AST 76 IU/L (normal: 10–60), and ALT 34 IU/L (normal: 10–35). EKG showed normal sinus rhythm. A urinary calcium/creatinine fractional excretion ratio was 0.03, and calcium/creatinine ratio was 0.9 at the time when serum calcium was 13.1 mg/dl. Both parents had normal serum calcium and phosphorous levels. Serum vitamin A level was 1738 mcg/L (normal: 360–1200 mcg/L). 

He was discharged home on the fifth hospital day with instructions for oral fluid intake of at least 1.5 L/m^2^/day. His total serum calcium on the day of discharge was 10.9 mg/dL. Serum calcium was to be checked by his primary physician daily and was stable for two days. Three days after discharge, he presented again to the emergency unit with recurrence of abdominal pain, vomiting, and generally not feeling well. He had not been able to keep up with the recommended oral hydration plan of at least 1.5 L/m^2^/day. At this point in time, mother also gave a history of hair loss since discharge. He had lost 2 kg in weight. He had marked blanching erythema on the cheeks and rest of the body together with cheilosis. Total calcium at this time was 12.6 mg/dL. He had not received any vitamin A since the initial admission seven days earlier. He was hospitalized once again and started on 3.0 L/m^2^/day normal saline hydration, 2 mg/kg/day of prednisone, and furosemide 1 mg/kg BID. Despite these measures, his calcium rose to 14.7 mg/dL during the next 24 hours (Table 2) at which point he was given a single dose of pamidronate 0.5 mg/kg IV. 

Calcium came down to 9.8 mg/dL over the next two days and to 8.4 mg/dL five days after pamidronate. He was changed to oral hydration, and serum calcium remained normal over the next few days. He was discharged home after six days to continue oral hydration and close followup. During this second admission, he had a skeletal survey that was remarkable only for a bilateral coxa valga deformity and mild generalized osteopenia. His serum vitamin A level was 2668 mcg/L. Tuberculin test was negative, and a chest X-ray and renal ultrasound were normal. Parathyroid hormone-related peptide (PTHrP) was 0.3 pmol/L (normal: <2 pmol/L). He also had a normal bone scan. After discharged home, he remained stable without additional hypercalcemia. His serum Vitamin A level 30 days after discontinuation of vitamin A was at 1201 mcg/L and serum calcium of 8.9 mg/dL.

## 3. Discussion

Hypercalcemia is an uncommon finding in children. Although uncommon, hyperparathryoidism can cause hypercalcemia in the pediatric age group [1]. In addition to hyperparathryoidism, the differential diagnosis of hypercalcemia includes malignancy, hypervitaminosis D, granulomatous diseases, medications, other endocrine disorders, and familial hypocalciuric hypercalcemia. The causes of the hypercalcemia are summarized in Table 3.

Our patient presented with nausea, abdominal pain, constipation, vomiting, and lethargy, all typical symptoms of hypercalcemia and his serum total calcium was 13.7 mg/dL. He underwent an extensive evaluation including careful history, family history, physical examination, and lab work to exclude possible causes of hypercalcemia. Serum phosphorus was normal, and plasma intact PTH was appropriately suppressed, ruling out hyperparathyroidism. 25-hydroxy vitamin D and 1,25-dihydroxy vitamin D levels were not elevated ruling out hypervitaminosis D. The low 1,25-dihydroxy-vitamin D probably reflects a reduced activity of the renal 1-hydroxylase enzyme activity in the face of suppressed PTH; PTH is required for activation of the 1-hydroxylase enzyme. Tuberculin test, chest X-ray, and bone survey were normal ruling out a granulomatous process (tuberculosis or sarcoidosis). Bone scan was normal and PTHrP were low, thus making malignancy-related hypercalcemia unlikely. Free T4 and TSH were normal, ruling out hyperthyroidism, and there were no signs or symptoms suggestive of adrenal insufficiency, acromegaly, or pheochromocytoma. Both parents had normal calcium and phosphorus and our patient's urinary calcium : creatinine ratio was high (0.9), thus ruling out familial hypocalciuric hypercalcemia (FHH). FHH results from heterozygous mutations in the calcium sensing receptor gene (*CASR*) and is inherited in autosomal dominant fashion. Clinically, patients with FHH have relative hypocalcuria (<4 mg/kg/day**) **and inappropriately normal PTH in the face of persistent mild hypercalcemia, none of which our patient had. He was not taking any medications known to cause hypercalcemia other then Vitamin A that he was receiving as part of his treatment for autism. Considering his history of excessive vitamin A intake (30,000 IU/day), his elevated vitamin A levels, and a complete evaluation to rule out other causes of hypercalcemia, this boy would appear to represent a case of hypercalcemia secondary to vitamin A intoxication. In addition, our patient had other signs of vitamin A intoxication, including alopecia, excoriation of skin, cheilosis and mild elevation in AST. 

Hypercalcemia as a complication of vitamin A therapy was first described in 1953 by Shaw and Niccoli [2]. We are aware of only 11 reported cases with this unique association of hypercalcemia as a complication of vitamin A toxicity. These reported cases fit into four general categories: (1) those receiving all-*trans* retinoic acid (ATRA) therapy for treatment of acute promyelocytic leukaemia [3]; (2) hemodialysis patients who consume nutritional supplements containing a pharmacological dosage of vitamin A [4]; (3) those who ingest massive dosage of vitamin A [5–10]; (4) those who become hypercalcemic secondary to vitamin A toxicity occurring during commercially available tube feeds [11]. To best of our knowledge, there has only been one other case report to date of a child with hypercalcemia secondary to vitamin A supplementation as part of a plan to improve the behavioral aspects of autism [12]. 

Vitamin A has a long biological half-life and accumulates in adipose tissue. The combination of relatively rapid absorption with slow clearance can produce acute toxicity after a high dose and chronic toxicity after prolonged intake of substantially smaller doses. Children are particularly sensitive to vitamin A and can become intoxicated with lower doses than those required to cause toxicity in adults. The toxic effects of vitamin A involve multiple organs. These organs include the bone, brain, liver, and the skin. Symptoms and signs include alopecia, anorexia, bone pain and tenderness, bulging fontanelles and craniotabes (in infants), fissuring of lip corners (cheilosis), hepatomegaly, hyperostosis, photophobia, pruritis, pseudotumour cerebri, skin desquamation, and skin erythema [13]. Prolonged vitamin A intoxication may cause premature epiphyseal closure. Our patient had many of these findings, including alopecia, anorexia, cheilosis, pruritis, skin desquamation and skin erythema.

It is not clear how vitamin A causes hypercalcemia and influences bone metabolism. Binkley and Krueger [14] reported hypercalcemia, elevated alkaline phosphatase, and an increased incidence of spontaneous bone fractures associated with hypervitaminosis A. Bélanger and Clark [15] found increased osteoblastic activity with an increase in periosteal bone apposition in rats fed large doses of vitamin A. This new bone was entirely cancellous. It was also noted that osteocytes matured rapidly in this new bone tissue compared to bone that had been previously laid down. Various degrees of enhanced osteolysis were recorded on *α*-radiographs. They concluded that under the influence of hypervitaminosis A, new bone was deposited and at the same time, greater resorption occurred. Fell and Mellanby [16] added pure vitamin A acetate (1000–3000 IU/100 mL) *in vitro *to tibia, fibulae, radii, and ulnae from 17- to 20-day old fetal mice and found that the matrix of terminal cartilage softened, shrank, and almost or completely disappeared whilst cartilage cells remained normal in appearance. This action was thought to be provoked by the release of bound protease and altered permeability of the lysosomes by the vitamin A. Though less dramatic than *in vitro*, these effects were confirmed *in vivo *[17]. In addition it is known that receptors for retinoic acid are located on both osteoclast and osteoblast [17]. All these findings suggest a direct effect of vitamin A on bone. 

The general goals for treatment of hypercalcemia include stabilization and reduction of the calcium level with adequate hydration, increasing urinary calcium excretion, inhibition of osteoclast activity in the bone, and treatment of the underlying cause (when possible). Specific therapeutic approaches include hydration, loop diuretics, glucocorticoids, calcitonin, and bisphosphonates. In cases of hypercalcemia secondary to vitamin A intoxication, several modes of therapy have been reported. Hydration with normal saline and diuresis with furosemide are common initial treatments. Glucocorticoids are often helpful. Wieland et al. (1971) [5] described use of prednisone 20 mg/kg to resolve hypercalcemia secondary to vitamin A toxicity (vitamin A level of 934) in a 16-year-old girl who had consumed 100,000 IU/day for 6 months. Frame et al. in 1974 [7] also described steroid use in a 46-year-old salesman with hypercalcemia and kidney stones who had a vitamin A level of 625 after consuming >75000 IU vitamin A per day. Prednisone 40 mg/day stabilized the calcium over one week; hypercalcemia recurred once the prednisone was stopped. Successful treatment of hypercalcemia secondary to hypervitaminosis A may require a combination of approaches and a treatment course that extends beyond the initial normalization of the calcium. This was true in our patient who promptly relapsed back to being hypercalcemic after the initial treatment appeared to have been successful.

Two cases have been reported of use of pamidronate in patients with vitamin A-induced hypercalcemia. Bhalla et al. [11] described a case of hypercalcemia secondary to excess vitamin A from enteral feeding. This patient was initially treated using aggressive hydration with normal saline and furosemide but as the serum calcium was persistently elevated, intravenous pamidronate was instituted and the hypercalcemia resolved. Sakamoto et al. [3] reported the case of an 11-year-old patient with known acute promyelocytic leukaemia who developed hypercalcemia after ATRA treatment. Pamidronate was used to treat the hypercalcemia. 

We report here a case of hypercalcemia secondary to vitamin A intoxication occurring during management of Autism. Autism is a spectrum disorder defined by DSM 4 criteria characterized by delay in language development, impairment of social interaction, and the use of restrictive stereotype behavioral patterns prior to three years of age. No single approach has proven effective in treating autism, and professionals and families have found that a combination of treatments may be effective in treating symptoms and behaviors that make it hard for individuals with autism to function. These may include psychosocial and pharmacological interventions. While there are no drugs, vitamins or special diets that have been clearly shown to correct the underlying neurological problems that seem to cause autism, changes to diet and the addition of certain vitamins or minerals are thought by some to help with behavioral issues. Over the past 10 years, there have been claims that adding essential vitamins such as B6 and B12 and removing gluten and casein from a child's diet may improve digestion, allergies, and sociability. Not all investigators and experts agree as to whether these therapies are effective or scientifically valid. It is also speculated that autism may be linked to a G-protein defect that affects the retinoid receptors in the brain. Therefore, vitamin A has been suggested as a possible intervention to improve visual and sensory perception, language processing, and attention [18]. In this paper, our patient was receiving 30,000 IU of vitamin A per day for 4 months; this is nearly ten times the recommended daily allowance (RDA) for vitamin A. Published literature states that RDA for vitamin A is 3000 IU in the pediatric age group and 5000 IU in adults [7]. There are no published data regarding the recommended dosage of vitamin A for treatment of autistic patients to help with the behavioral and neurological aspect of the condition. 

Hypercalcemia is a potentially serious side effect of vitamin A supplementation or retinoic acid treatment for various disorders. In cases of hypercalcemia, it is important to obtain a careful dietary and medication history to assist in identifying the underlying etiology. In addition, patients treated with vitamin A, retinoic acid, or their analogs should be monitored for serum calcium levels at regular intervals. When hypercalcemia complicates the therapy, consideration should be given to discontinuing vitamin A supplementation or retinoic acid treatments unless clear benefit of such treatments has been observed.

## Figures and Tables

**Table 1:** Course of calcium levels during first hospital stay.

Day	Time	Total calcium	Comments
1	19:37	13.7	IV hydration 3.0 L/m^2^/day **+ **furosemide 1 mg/kg IV
2	04:01	13.1	
2	17:14	12.7	
2	23:14	11.5	IV hydration reduced to 1.5 L/m^2^/day
3	05:00	11.4	Oral rehydration started
3	17:55	10.6	
4	05:21	10.0	
4	17:43	10.2	
5	11:38	10.9	Discharged home

**Table 2:** Course of calcium levels during second hospital stay.

Day	Time	Total Calcium	Comments
1	15.47	12.6	IV hydration 3.0 L/m^2^/day + Prednisone 2 mg/kg/day + Furosemide 1 mg/kg BID
2	08:00	12.5	
2	23:00	14.7	Pamidronate 0.5 mg/kg IV
			IV hydration 3.5 L/m^2^/day
3	06:24	13.4	
4	16:30	11.4	Oral rehydration started
4	22:15	10.8	
5	08:05	9.8	
6	10:00	9.5	
7	10:00	8.4	Discharged home

**Table 3:** Causes of hypercalcemia.

*Parathyroid hormone-related *
Primary hyperparathyroidism
Sporadic
Familial
MENI or MENII
Tertiary hyperparathyroidism
Associated with renal insufficiency
*Vitamin D-related *
Vitamin D intoxication
Granulomatous disease
*Malignancy *
Humoral hypercalcemia of malignancy
(Associated with PTHrP)
Solid tumors
*Medications *
Thiazide diuretics
Lithium
Milk-alkali syndrome
Vitamin A intoxication
*Other endocrine disorders *
Hyperthyroidism
Adrenal insufficiency
Acromegaly
Pheochromocytoma
*Familial hypocalciuric hypercalcemia (FHH)*:
(Mutation in calcium-sensing receptor)
